# Mind the gap: an administrative data analysis of dental treatment outcomes and severe mental illness.

**DOI:** 10.23889/ijpds.v7i3.2001

**Published:** 2022-08-25

**Authors:** Finola Ferry, Michael Rosato, Gerard Leavey

**Affiliations:** 1 Ulster University

## Objectives

Oral health of people with severe mental illness (SMI) remains an important public health issue with some evidence pointing suboptimal dental health outcomes. We test the hypotheses that individuals with SMI have lower contact with dental services and higher levels of fillings and extractions. We also examine effect modification by age.

## Approach

We used linked administrative data from general practitioner (GP) records, hospital admissions data and dental payment system data to examine dental service use and treatments (extractions, fillings, crowns and x-rays) among the Northern Ireland hospital population between January 2015 and November 2019 (N=798,564). Logistic regression examined the association of SMI with dental service use and treatments, controlling for socio-demographic characteristics. In a further series of models, the interaction of SMI and age-group was examined.

## Results

An estimated 1.2% (9,656) recorded an SMI diagnosis from hospital records at some point during follow-up. Over two-thirds (67.2%) of the non-SMI hospital population received any dental treatment compared with 62.2% of persons SMI-diagnosed. After adjusting for available socio-demographic characteristics, analysis indicated lower levels of dental service use (OR=0.80, 95% CI=0.77, 0.84), including lower likelihood of fillings (OR=0.81, 0.77, 0.84) and x-rays (OR=0.77, 0.74, 0.81), but higher levels of extractions (OR=1.23, 1.18, 1.29) among patients with SMI. We also found effect modification by age-group: while older age was generally associated with higher likelihood of extractions and crowns, interaction analysis points to lower likelihood of all four dental treatments among older individuals with SMI.

## Conclusion

Our findings of lower engagement with dental services, lower levels of remedial treatments and higher levels of tooth loss raise concerns about availability, access, and promotion of routine dental care for people with SMI. There is a need for improved understanding of barriers to treatment and development of psychoeducational interventions.

